# A comparison of symptom dimensions for obsessive compulsive disorder and obsessive compulsive-related disorders

**DOI:** 10.1371/journal.pone.0218955

**Published:** 2019-07-05

**Authors:** Derya Say Öcal, Kadir Özdel, Yasir Şafak, Yasemin Kekilli Karnaz, Cebrail Kısa

**Affiliations:** 1 Department of Psychiatry, Kecioren Training and Research Hospital, Ankara, Turkey; 2 Department of Psychiatry, Diskapi Yildirim Beyazit Training and Research Hospital, Ankara, Turkey; 3 Department of Psychiatry, Gaziantep Nizip State Hospital, Gaziantep, Turkey; 4 Department of Psychology, İstanbul Aydın University, İstanbul, Turkey; eCampus University, ITALY

## Abstract

**Objective:**

In this study, it is aimed to determine obsessive compulsive-related disorders (OCRDs) comorbidity among the patients with obsessive compulsive disorder (OCD) and compare patients with OCD with or without comorbid OCRDs in terms of the severity of their OCD symptoms, symptom dimensions, and comorbidity with other axis I disorders.

**Methods:**

The study included 90 patients diagnosed as having OCD according to the Diagnostic and Statistical Manual of Mental Disorders, Fourth Edition, Text Revision (DSM-IV-TR). The Diagnostic and Statistical Manual of Mental Disorders, Fifth Edition (DSM-5) diagnostic criteria for OCRDs were used to determine the presence of OCRDs. In order to determine the symptom dimensions and severity of these individuals’ OCD symptoms, we administered the Dimensional Obsessive Compulsive Scale (DOCS) and The Yale-Brown Obsessive Compulsive Scale (Y-BOCS).

**Results:**

In our study, 20% of the patients with OCD simultaneously met the criteria for at least one OCRD, we also found that a significantly greater proportion of this group were men. None of the mentioned disorders was associated with any symptom dimensions we evaluated using DOCS. In addition, no differences were found in the severity of OCD symptoms and comorbid axis I disorders between the group with comorbid OCRDs and the group without comorbid OCRDs.

**Discussion:**

There was no significant relationship between the symptom dimensions of OCD and OCRDs. It is found that OCRD comorbidity does not increase the severity of OCD symptoms and the prevalence of an axis I diagnosis.

## Introduction

Obsessive-compulsive disorder (OCD) is characterized by the presence of obsessions, compulsions or both. The American Psychiatric Association (2013) defines obsessions as recurrent, persistent and unwanted thoughts, urges or images that cause marked anxiety and distress, and that individuals try to ignore, suppress or neutralize their obsessions with other thoughts or actions. Compulsions are repetitive behaviors or mental acts that a person feels driven to perform in response to obsessions, performed according to rules that must be strictly obeyed.[1]

According to the Diagnostic and Statistical Manual of Mental Disorders, Fourth Edition (DSM-IV), at least one axis I psychological disorder is comorbid in 27–92% of patients with OCD. [2–5] However, in many studies, life-time comorbidity for OCD ranges between 60% and 90%. [3, 5–9] The disorders most commonly comorbid with OCD are depressive disorders and anxiety disorders. [3–8, 10] Common comorbid obsessive–compulsive spectrum disorders with OCD are body dysmorphic disorder (BDD), trichotillomania, skin picking, and tic disorders. [3, 8–10]

The relationship between OCD and OCD spectrum disorders has been the focus of considerable debate. OCD used to be classified within ‘Anxiety Disorders’ in DSM-IV-TR. [11] In the Diagnostic and Statistical Manual of Mental Disorders, Fifth Edition (DSM-5) published in 2013 by the American Psychiatric Association, OCD is now classified with ‘OCD and Related Disorders’ as a separate diagnostic category. [1] The disorder’s primary symptoms involve repetitive behaviors and a subjective sense of compulsion. OCD and OCD spectrum disorders have common features such as etiology, comorbidity, brain circuits, neurotransmitter abnormalities, family history, hereditary characteristics, and responses to treatment. [12]

Although many studies have debated the relationship between OCD and OCD spectrum disorders, a number of studies have systematically investigated the comorbidity of OCD spectrum disorders in patients with OCD. One of these studies showed that 57.6% of patients with OCD concurrently met the criteria for at least one OCD spectrum disorder and 67.1% had at least one coexistent OCD spectrum disorder in their lifetime history. [13] As a result of the studies that described a relationship between OCD and tic disorder and Tourette’s syndrome [14–16], the DSM-5 included a *tic-related specifier* for OCD. Studies to date have shown that the prevalence of co-occurring BDD is 8.7–14.5% [10, 17, 18], the prevalence of trichotillomania is 5–9% [19–21], and the prevalence of skin-picking (excoriation) is 7.8–16.3% [22, 23] for patients with OCD. Relatives of patients with OCD are also frequently diagnosed as having tic disorders, trichotillomania, BDD, hypochondriasis, and other disorders such as eating disorders and nail biting. [16, 20, 24]

Different individuals show a high level of heterogeneity in their symptoms of OCD. [25, 26] Since OCD was first identified, researchers have tried to separate symptoms into homogeneous subtypes. [27–32] Each subtype may reflect different underlying psychopathologic processes related to different genetic transition mechanisms and environmental impacts. [33] They may also differ in terms of response to treatment and psychiatric comorbidity [34, 35] and have different neuroimaging symptom profiles. [36, 37] Studies of the symptom dimensions of OCD have found a relationship between the symmetry/order dimension and tics and obsessive-compulsive personality disorder. [29, 38] The hoarding dimension has been described as related to male sex, early onset, impaired social functionality, personality disorders, low response to selective serotonin reuptake inhibitors (SSRI), a high ratio of quitting treatment and to other anxiety disorders. [35] A relationship has been found between female sex and contamination/cleaning dimensions, and between male sex and sexual and religious obsessions. [39] Although patients who respond negatively to SSRI treatment frequently show hoarding/symmetry dimensions and sexual and religious obsessions, their responses vary for cognitive behavioral therapy. [40]

The purposes of this study were to examine obsessive compulsive-related disorders (OCRDs) comorbidity in OCD patients and to compare OCD patients with and without OCD-related disorders in terms of their symptom dimensions and to investigate whether it was more common for patients with OCD-related disorders to have certain obsessions and compulsions. The main hypothesis of this study was that patients with OCD with other OCD-related disorders would frequently have more of certain obsessions and compulsions, and that obsessive-compulsive symptoms tended to be more severe for these patients.

## Methods

### Sample

Our study sample included 90 consecutive patients aged over 18 years who were admitted to the Ankara Diskapi Yildirim Beyazit Training and Research Hospital Outpatient Service between October 2013 and April 2014. All these patients were newly diagnosed or had been diagnosed earlier as having OCD according to the DSM-IV-TR diagnostic criteria. The study was reviewed and approved by Diskapi Yildirim Beyazit Training and Research Hospital ethics committee. Written consents of the patients were obtained after informing them about the study. Exclusion from the study was based on intellectual disability, cognitive mental disorders, psychotic disorders, bipolar mood disorder, or insufficient education to complete the study tests.

### Procedures

Diagnoses of patients included in the study were made using the Structured Clinical Interview Form (SCID-I) for DSM-IV Axis I Disorders. The patients were assessed according to DSM-5 diagnostic criteria for OCD-related disorders. Severity of OCD symptoms was assessed using the Yale-Brown Obsession Compulsion Scale (Y-BOCS). The Dimensional Obsession Compulsion Scale (DOCS) was used to assess the symptom dimensions of OCD.

This group of patients with OCD was examined in terms of their sociodemographic characteristics, comorbidity, severity of OCD symptoms, and symptom dimensions. Patients with a comorbid diagnosis with OCD-related disorders were then compared with patients with no co-occurring diagnoses, using the same parameters.

### Measures

#### Sociodemographic information form

This form was given to the patients to query their sociodemographic characteristics and the clinical features of their disorder.

#### The structured clinical interview for DSM-IV axis I disorders

This is a semi-structured clinical interview tool applied by a trained interviewer in order to investigate axis I psychiatric disorder diagnoses for patients according to DSM-IV. [41] The Turkish validity and reliability study was established by Özkürkçügil et al. [42]

#### The Yale-Brown Obsessive Compulsive Scale-Y-BOCS

The Turkish validity and reliability of this scale, which was developed in 1989 by Goodman et al. [43], was established by Karamustafalıoğlu et al. [44] The scale consists of a total of 19 items and is administered by an interviewer. The first 10 items are used to determine the total score. In scoring, each of the subscales of obsession and compulsion has five items and each item is individually scored between 1 and 4. The total score of the subscales for obsession and compulsion provide an overall maximum score of 40.

#### The Dimensional Obsessive Compulsive Scale (DOCS)

The Dimensional Obsessional Compulsive Scale (DOCS) is a 20-item self-report scale developed by Abramowitz et al. [26] This scale evaluates four distinct obsessive compulsive symptom dimensions; each scale contains general definitions and includes examples of avoidant behavior measures and assesses the severity of each symptom dimension. Thus, the scale provides a link between obsessive, compulsive, and avoidance behaviors in each symptom dimension, and measures the severity of symptoms independently of the types of obsessions and compulsions present. The most frequent identified OCD symptoms in this structural analysis included 1) contamination, 2) responsibility for harm or mistakes, 3) incompleteness, and 4) unacceptable thoughts. Rather than listing the specific indications contained in each dimension, a number of typical thought examples, rituals, and compulsions are presented for each dimension. Within each symptom dimension there are 5 items (to be answered with regard to the prior 30 days), which measure severity according to various parameters. These items are a) time spent with the symptoms, b) avoidance behavior, c) distress experienced, d) impairment/deterioration in functionality, and e) difficulty in coping with the symptoms. The scale is formed from 4 dimensions, each having five items scored between 0 and 4. The Turkish validity and reliability study of this scale was determined by Şafak et al. [45]

### Statistical analysis

All data were analyzed by using SPSS Windows version 16.0. Quantitative data are specified as percentages, averages and standard deviations. T-test was used for independent samples to compare normally distributed parameters of two groups. Bonferroni correction was used to determine the value of significance, and the p-value was set as .01 with this correction. On the other hand, two groups were compared on the parameters that were not normally distributed by using the Mann-Whitney U test. Additionally, Pearson’s Chi-square test was used for analyzing relationships between categorical variables. Finally, the significance level was determined as *P* < .05.

## Results

Of the patients included in this study (N = 90), 64 (71.1%) were female and 26 (28.9%) were male. Their ages ranged between 18 and 52 years (mean: 27.4±8.1 years). The distribution of other sociodemographic characteristics of the patients is given in S1 Table.

Twenty-one OCD-related disorders were found in 18 (20%) of the patients. The distribution of these disorders are shown in Table 1.

**Table 1:** Distribution of disorders related to comorbid obsessive-compulsive disorder.

Comorbidity	n	%
***Trichotillomania***	5	5.6
***Skin picking disorder***	9	10
***Body dysmorphic disorder***	4	4.4
***Hoarding disorder***	3	3.3
***Total***	21	23.3

Pearson’s Chi-square test was used to compare the distribution of the comorbid OCD-related disorders between the sexes. OCD-related disorders were found significantly more frequently in male patients with OCD (*P* = .027) (Table 2).

**Table 2:** Distribution of obsessive-compulsive disorder-related disorders among the sexes.

		Female	Male	*P*
**n = 18**		9	9	
**OCD-related disorder present**	Among OCD-related disorder (%)	50	50	
** **	Among sexes (%)	14.1	34.6	.027*
**n = 72**		55	17	
**OCD-related disorder not present**	Among OCD-related disorder (%)	76.4	23.6	
** **	Among sexes (%)	85.9	65.4	

OCD = Obsessive Compulsive Disorder.

*Statistically significant at the level of p < .05.

The samples’ Y-BOCS and DOCS scores were assessed using a t-test to compare OCD severity levels and symptom dimensions of patients with OCD with and without comorbid OCD-related disorders (Table 3). Bonferroni correction was performed to determine the significance value. No difference was found between the groups in terms of their Y-BOCS and DOCS scores.

**Table 3:** Comparison of the Y-BOCS and DOCS scores of the patients with and without comorbid OCD-related disorders.

	OCD-related Disorder	n	Mean	S.D.	p*
**YBOCS Total**	Yes	22	25.5	7.4	.459
	None	68	24.2	6.9	
**DOCS Category 1**	Yes	22	7.1	5.6	.844
	None	68	7.4	6.1	
**DOCS Category 2**	Yes	22	6.9	5.9	.986
	None	68	6.8	5.7	
**DOCS Category 3**	Yes	22	7.2	5.4	.825
	None	68	7.5	6.7	
**DOCS Category 4**	Yes	22	4.6	4.6	.224
	None	68	6.2	5.6	

OCD = Obsessive Compulsive Disorder, Y-BOCS = Yale-Brown Obsessive Compulsive Scale, DOCS = Dimensional Obsessive Compulsive Scale.

*Bonferroni correction *p* value: (0.05/5) = .01.

When the patients were assessed for SCID-I comorbidities according to DSM-IV, no axis I diagnoses were found in 34 (37.8%) patients, and at least one axis I diagnosis was found for 56 (62.2%) patients. Major depression was found in 27 (30%) patients, simple phobias in 10 (11.1%) patients, generalized anxiety disorder (GAD) in 9 (10%) patients, social phobia in 8 (8.8%) patients, panic disorder in 5 (5.6%) patients, and dysthymia in 4 (4.4%) patients.

The patients we studied with OCD-related disorders were also assessed in terms of SCID-I comorbidities according to the DSM-IV. No axis I comorbidity was found in 9 of 18 patients, major depression was found in 8 patients, social phobia in 3 patients, simple phobia in 2 patients, and panic disorder in 1 patient. The patients with and without comorbid OCD-related disorders were compared using Pearson’s Chi-square test in terms of SCID-I comorbidities according to the DSM-IV. No significant difference was observed between the two groups in terms of axis I comorbidities (*P* = .913) (Table 4).

**Table 4:** Comparison of the patients with and without obsessive-compulsive disorder-related disorders in terms of axis I comorbidities.

			Axis I Comorbidity Present	Axis I Comorbidity Not Present	*P*
**OCD-related disorder**	n		11	7	.913
	**Yes**	%	61.1	38.9	
	**None**	n	45	27	
		%	62.5	37.5	
**Total**			56	34	
		%	62.2	37.8	

OCD = Obsessive Compulsive Disorder.

Statistically significant at the level of *p* < 0.05.

## Discussion

Our study aimed to examine obsessive compulsive-related disorders (OCRDs) comorbidity in OCD patients and to compare OCD patients with and without comorbid OCD-related disorders in terms of symptom severity, comorbidities, and symptom dimensions. We developed two essential hypotheses related to existing scientific data and observations. Our first hypothesis was that some obsessions and compulsions were more frequently found in patients with OCD with certain comorbid disorders specified under OCD-related disorders. The second hypothesis was that obsessive-compulsive symptoms were more severe in these same patients.

When we examined the patients in terms of comorbid OCD-related disorders, we determined that at least one of these disorders was present in 20% of patients. Skin picking disorder was observed in 10%, trichotillomania was found in 5.6%, BDD was found in 4.4%, and hoarding disorder was found in 3.3% of patients. In studies of patients with OCD, the incidence of comorbid trichotillomania varies between 5% and 9% [19–21], and the ratio of those with BDD varies between 8.7% and 14.5%. [10, 17, 18] The frequency of trichotillomania found in our study is in agreement with the literature and the rates of comorbid BDD disorder in our patients with OCD were not as high as rates found in other studies. When we take into consideration that a diagnosis of BDD is generally made in patients’ in their mid-thirties, the low rates of BDD ratio found in this study may be associated with the low average age of our study patients. In studies performed to date, hoarding behavior was found in 8.6–40% of patients with OCD. [7, 39] Hoarding behavior causes serious distress in only 5.5% of these patients. The rates specified in these studies were obtained by assessing hoarding as a symptom, not a disorder. In our study, patients were assessed according to the hoarding disorder diagnosis criteria in the DSM-5 and it is found the prevalence of the disorder as 3.3%. A limited number of studies have examined skin picking disorder and a few studies have investigated the symptom under the heading of impulse control disorders. Skin picking occurs in patients with OCD at a rates between 7.8% and 16.3%. [22, 23] The rates found in our study are in agreement with the literature. According to our search, our study is one of the very first studies that investigate the prevalence of DSM-5 OCDRs in patients with OCD.

When we assessed patients with comorbid OCD-related disorders in terms of their sex, the disorders we considered were found significantly more frequently in male patients. In a study by Du Toit et al., 85 patients with OCD were assessed in terms of OCD spectrum disorders (eating disorders, BDD, impulse control disorders and depersonalization disorder) and they found that patients with comorbid spectrum disorders were mostly female. [13] The fact that Du Toit et al. included eating disorders, which are known to be more common in women, may also have caused this difference. Other studies in the literature that considered trichotillomania, skin picking disorder, and BDD have varied more in relation to sex prevalence. [46, 47] Our study cannot fully analyze the relationship between sex and specific OCD-related disorders due to the relatively small sample of such patients. As seen in our study OCRDs are seen frequently in patients who come to the outpatient clinic with OCD symptoms. Therefore, we suggest that it is important to screen OCD-related symptoms in these patients. Since it is found that OCD-related disorders are significantly more frequent in male patients in our study, we think it would be helpful to ask for these diseases without gender difference.

In our study, as Du Toit et al. also found [13], no significant differences were found in severity between patients OCD with and without comorbid OCD-related disorders. This suggests that OCD-related disorders that accompany OCD do not affect the severity of the disorder, and obsessions and compulsions may be independent of these disorders.

Our study showed no relationship between OCD-related disorders and any particular symptom dimensions of OCD. There was no difference in symptom dimensions between the group with OCD only and the group with comorbid OCD-related disorders.

When the patients were assessed in terms of comorbidities, at least one axis I diagnosis was observed in 62.2% of the patients, and major depression was the most frequent comorbid psychiatric disorder at a rate of 30%. These results are generally in agreement with the literature. [2–8] However, when patients with OCD with and without concomitant OCD-related disorders were compared in terms of their DSM-IV axis I comorbidities, no significant differences were observed between the groups.

Based on the results of our study, when patients with OCD with and without comorbid OCD-related disorders were assessed together, no relationship was evident between the severities of their OCD symptoms. There was no difference in their axis I comorbidity rates and no relationship between their OCD-related disorders and any of several OCD symptom dimensions. This study contributes to the literature in this area, which we expect will be examined further based on the DSM-5.

One of the limitations of our study is that the YBOCS and DOCS scores of treated and newly diagnosed patients with OCD may vary depending on how long they have been treated and whether they show any treatment response. The patients who participated in our study were currently receiving treatment and some were just beginning treatment. The most important limitation of our study is the limited number of patients, which makes it difficult to compare various OCD-related disorders. In larger samples the distribution of symptoms and statistical validity of the analysis would be greater. Based on both these factors, studies with larger samples using a healthy control group are needed.

## Supporting information

S1 Table Distribution of sociodemographic characteristics of the participants. (DOCX)

S1 Data The data collected and used for the study. (XLSX)
