# Supplementary material for: A comparison of symptom dimensions for obsessive compulsive disorder and obsessive compulsive-related disorders
Source: PLoS One. 2019 Jul 5;14(7):e0218955. doi: 10.1371/journal.pone.0218955 (PMC6611656; doi:10.1371/journal.pone.0218955)
Supplement: S1 Table — (DOCX) [file pone.0218955.s001.docx]

**S1 Table:** Distribution of sociodemographic characteristics of the participants

|  |  | **Min-Max** | **Mean±SD** |
| --- | --- | --- | --- |
| **Age (years) (n=90)** |  | 18-52 | 27.4±8.1 |
|  |  | **n** | **%** |
| **Sex** | Female | 64 | 71.1 |
|  | Male | 26 | 28.9 |
| **Marital status** | Single | 56 | 62.2 |
|  | Married | 34 | 37.8 |
| **Educational Status** | Primary school | 18 | 20.0 |
|  | Secondary school | 10 | 11.1 |
|  | High School | 39 | 43.3 |
|  | College | 23 | 25.6 |
| **Occupational Status** | Unemployed | 15 | 16.7 |
|  | Student | 35 | 38.9 |
|  | Housewife | 23 | 25.6 |
|  | Worker/Civil servant | 17 | 18.9 |
| **Cohabitants** | Spouse and children | 28 | 31.1 |
|  | Parents | 41 | 45.6 |
|  | Single | 4 | 4.4 |
|  | Extended family | 14 | 15.6 |
|  | Relatives | 3 | 3.3 |
| **Income level** | Low | 31 | 34.4 |
|  | Medium | 29 | 32.2 |
|  | High | 30 | 33.3 |
| **Place where grew up** | Rural | 18 | 20.0 |
|  | Urban | 72 | 80.0 |
| **Living place** | Rural | 4 | 4.4 |
|  | Urban | 86 | 95.6 |
|  |  | **Min-Max** | **Mean** |
| **No. of siblings** |  | 0-10 | 3.4 |
|  |  |  |  |
